# Caspase-1 cleavage of transcription factor GATA4 and regulation of cardiac cell fate

**DOI:** 10.1038/cddis.2014.524

**Published:** 2014-12-11

**Authors:** A Aries, J Whitcomb, W Shao, H Komati, M Saleh, M Nemer

**Affiliations:** 1 Molecular Genetics and Cardiac Regeneration Laboratory, Departments of Biochemistry, Microbiology, and Immunology, University of Ottawa, 550 Cumberland, Room 246, Ottawa, Ontario K1N 6N5, Canada; 2 Departments of Medicine and Biochemistry, McGill University, 3648 Sir William Osler Promenade, Room 364, Montréal, Québec H3G 0B1, Canada

## Abstract

Caspase-1 or interleukin-1*β* (IL-1*β*) converting enzyme is a pro-inflammatory member of the caspase family. An IL-1*β*-independent role for caspase-1 in cardiomyocyte cell death and heart failure has emerged but the mechanisms underlying these effects are incompletely understood. Here, we report that transcription factor GATA4, a key regulator of cardiomyocyte survival and adaptive stress response is an *in vivo* and *in vitro* substrate for caspase-1. Caspase-1 mediated cleavage of GATA4 generates a truncated protein that retains the ability to bind DNA but lacks transcriptional activation domains and acts as a dominant negative regulator of GATA4. We show that caspase-1 is rapidly activated in cardiomyocyte nuclei treated with the cell death inducing drug Doxorubicin. We also find that inhibition of caspase-1 alone is as effective as complete caspase inhibition at rescuing GATA4 degradation and myocyte cell death. Caspase-1 inhibition of GATA4 transcriptional activity is rescued by HSP70, which binds directly to GATA4 and masks the caspase recognition motif. The data identify a caspase-1 nuclear substrate and suggest a direct role for caspase-1 in transcriptional regulation. This mechanism may underlie the inflammation-independent action of caspase-1 in other organs.

Caspase-1 is best known for its role in inflammation through the processing of the pro-inflammatory cytokines interleukin-1*β* (IL-1*β*) and IL-18.^1^ Mice lacking caspase-1 (*Casp1*^*−/−*^) are viable but fail to activate and secrete IL-1*β*.^2^ In addition to its function in inflammation, caspase-1 has a role in programmed cell death in myeloid cells, lymphocytes as well as in the heart and brain.^3, 4^ Whereas *Casp1*^*−/−*^ mice have no developmental programmed cell death defects, they are protected against ischemic brain injury and heart failure.^3, 4^ In both neurons and cardiomyocytes, a direct role for caspase-1 in promoting cell death in the absence of inflammation has been demonstrated using *in vitro* cell cultures and *in vivo* models. For example, myocardial-specific overexpression of caspase-1 induces a massive increase in cardiomyocyte death in young mice without any increase in tissue or plasma levels of IL-1*β*, IL-18 or other inflammatory mediators; conversely, *Casp1*^*−/−*^ mice show a lesser degree of cell death after induction of myocardial infarction.^4^ Similarly, expression of caspase-1 in neonate rat cardiomyocyte cultures increases cell death by 4- to 5-fold.^4^ Because postnatal cardiomyocytes have limited regenerative capacity, their loss as occurs following myocardial infarction or chemotherapy leads to heart remodeling, loss of contractility and ultimately heart failure.^4^ Indeed, cardiomyocyte death is increased in human heart failure and induction of cell death in experimental models is sufficient to cause heart failure.^5^ Together, the data suggest that caspase-1 inactivates key molecules and pathways that promote cardiomyocyte survival.

Transcription factor GATA4, a member of the zinc finger GATA family, has emerged as a key cardiomyocyte survival factor and an essential regulator of the postnatal cardiomyocyte stress response. Cardiomyocytes with downregulated GATA4 levels have increased rates of cell death at basal levels and in response to cardiotoxic drugs such as Doxorubicin (Dox) or tyrosine kinase inhibitors.^6, 7^ These cells also fail to mount any adaptive response to mechanical or neuroendocrine stress.^8, 9, 10^ GATA4 is also a potent cardiogenic factor essential for cardiomyocyte commitment and differentiation.^11^ We now report that GATA4 is cleaved by caspase-1 *in vitro* and in cardiomyocytes. The resulting cleaved protein acts as a dominant negative isoform unable to maintain the genetic program required for myocyte survival. The data identify a target for caspase-1 in the nucleus and a pathway to explain its cardiac action.

## Results

**Figure 1:**
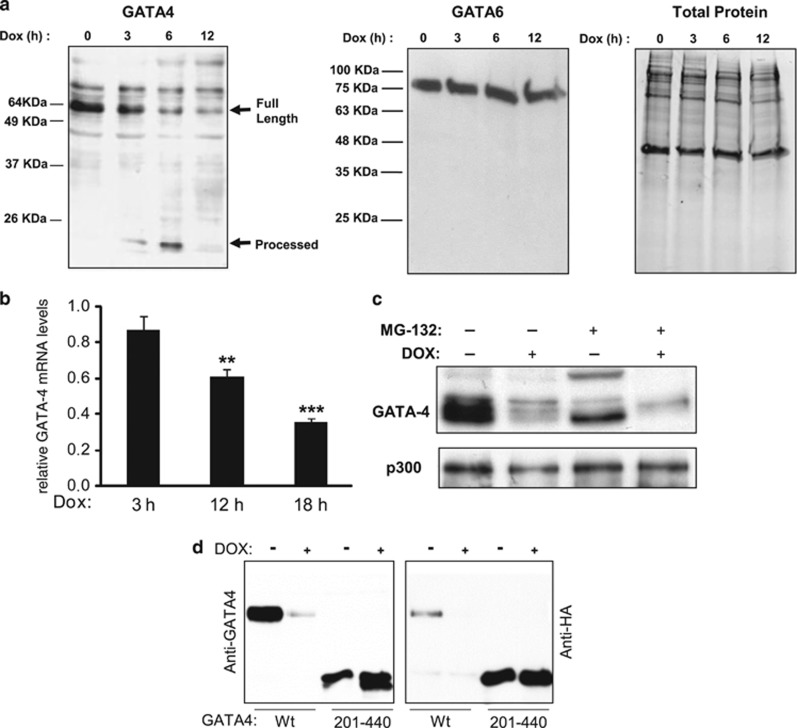
Dox-induced GATA4 depletion is independent of the ubiquitin-proteasome pathway. (**a**) Effect of time course treatment of Doxorubicin (Dox) on GATA4 (left panel), GATA6 (middle panel) and total protein (right panel) levels. Nuclear extracts were prepared from primary cardiomyocyte cultures treated for the indicated times with Dox (300 nM) and subjected to western blot analyses. (**b**) Depletion of GATA4 transcripts after 12 h of Dox treatment. Cardiomyocytes were treated for the indicated times with Dox. RNA was subjected to real-time PCR. GATA4 mRNA levels were normalized to S16 mRNA. The results are shown as mean±S.E.M. and analyzed by one-way ANOVA with Bonferroni post-test relative to the 3-h Dox treatment (*n*=3). **P≤0.01, ***P≤0.001. (**c**) Depletion of GATA4 protein by Dox is not prevented by a proteasome inhibitor. Cardiomyocytes were treated with Dox for 12 h in the presence or absence of 10 *μ*M proteasome inhibitor MG132. Nuclear extracts were subjected to western blot to detect GATA4 protein. P300 was used as a control. (**d**) A putative cleavage site in the N-terminal region of GATA4. Transient transfection was carried out in HL-1 atrial cardiomyocytes using GATA4 WT and a GATA4 N-terminal deletion (201–440) mutant. Nuclear extracts were subjected to western blot analysis using anti-HA and anti-GATA4 antibodies to detect N- and C-terminal fragments, respectively

GATA4 is an immediate early target of Doxorubicin (Dox) in the heart, affecting both transcriptional and post-translational mechanisms. Depletion of GATA4 dose dependently induces cell death, a process that can be rescued by exogenous GATA4.^6^ Time course analysis of Dox effects revealed that the GATA4 protein was markedly depleted after 3 h of treatment (the earliest point studied) in the absence of any significant decrease in transcript levels (Figures 1a, left panel and b). GATA6 protein levels remained unchanged (Figure 1a, middle panel). The decrease in the native GATA4 immunoreactive band was accompanied by the concomitant appearance of a 20-KDa band. GATA4 degradation was independent of the proteasome as shown by the inability of a proteasome inhibitor to prevent the Dox-dependent decrease in GATA4 protein (Figure 1c). To confirm whether these changes occur at post-translational stages, a CMV-driven HA-GATA4 expression vector was transfected into the cardiomyocyte cell line HL-1 and treated with Dox. As shown in Figure 1d, Dox-treated extracts had significantly less intact exogenous GATA4 as revealed with the HA and GATA4 antibodies which recognize N and C-terminal epitopes, respectively.^12^ A GATA4 protein deleted of its entire N-terminal domain (amino acids 201–440) was then transfected into HL1 cells and exposed to Dox. In Dox-treated cells, the C-terminal GATA4 antibody detected a doublet suggesting that a cleavage site lies within this domain. This doublet was not recognized by the N-terminal HA tag implicating cleavage at the N-terminus of the protein. The difference in size between the two bands suggested cleavage between amino acids 225 and 230.

**Figure 2:**
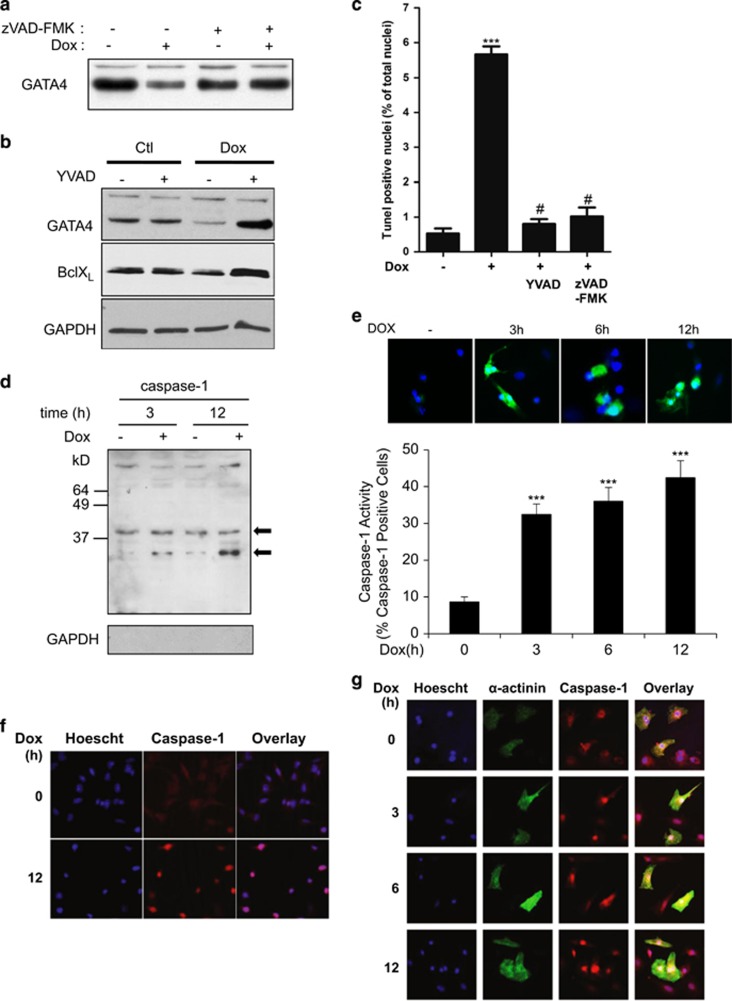
Dox-induced GATA4 depletion is caspase-1 dependent. (**a**) Pan-caspase inhibitor restored GATA4 expression. Cardiomyocytes were treated with Dox in the presence or absence of pan-caspase inhibitor (zVAD-FMK) for 12 h and analyzed by western blot. (**b**) Caspase-1 inhibitor prevented Dox-dependent GATA4 depletion. Cardiomyocytes were treated in the presence or absence of Dox with a caspase-1 inhibitor (YVAD-CHO). Western blots were carried out to detect GATA4 and its downstream target BclxL. GAPDH was used as a loading control. Note how changes in BclxL levels parallel those of GATA4. (**c**) Effect of caspase inhibition on cardiomyocyte apoptosis. Quantification of TUNEL assays in primary cardiomyocytes treated with the indicated inhibitors. The results are shown as mean±S.E.M. and analyzed by one-way ANOVA with Bonferroni post-test relative to the control (*) or to the Dox treatment alone (^#^). ***P≤0.001, ^#^*P*≤0.001. Note how caspase-1 inhibition is as effective as the pan-caspase inhibitor at abrogating Dox-induced apoptosis. (**d**–**g**) Increased activation and nuclear localization of caspase-1 in Dox-treated cardiomyocytes. (**d**) Western blots of nuclear cardiomyocyte extracts. Notice how caspase-1 is activated (lower band) after 3 and 12 h of Dox treatment. GAPDH staining was used to control for cytoplasmic contamination. (**e**) Representative images (top panel) and quantification (lower panel) of a FAM-FLICA assay measuring caspase-1 activity in control and Dox-treated cardiomyocytes. Results are shown as percent of caspase-1-positive cells. ***P≤0.0001. In the top panel, green is active caspase-1 and blue is Hoechst staining. (**f**) and (**g**) Immunofluorescence of HL1 cells (**f**) and primary cardiomyocytes (**g**) treated with Dox for the indicated time. Caspase-1 is labeled in red, α-actinin is labeled in green and Hoechst staining is labeled in blue

We tested whether GATA4 degradation was caspase dependent. Co-treatment of cardiomyocytes with Dox and a pan caspase inhibitor abrogated GATA4 depletion (Figure 2a) and significantly attenuated cardiomyocyte death (Figure 2c). Next, we determined which caspase was responsible for GATA4 depletion and cardiomyocyte death. Cardiomyocytes were treated with Dox in the presence or absence of YVAD-CHO, a selective caspase-1 inhibitor. Co-treatment with Dox and YVAD-CHO prevented GATA4 depletion, demonstrating that inhibition of caspase-1 protects against Dox-induced GATA4 degradation (Figure 2b). In contrast, caspase-3 inhibition had only a modest effect on GATA4 levels and on Dox-induced cardiomyocyte apoptosis (data not shown). The caspase-1 inhibitor was also as effective as the pan-caspase inhibitor at reducing cell death in response to Dox treatment (Figure 2c). These results are indicative of an important role for caspase-1 in Dox-induced cardiotoxicity. We examined whether Dox treatment was associated with caspase-1 activation by both western blot and FLICA assay, which measures active caspase-1 binding to cognate sites. Western blot analysis of nuclear extracts revealed the presence of cleaved caspase-1 in Dox-treated cardiomyocytes at 3 and 12 h post treatment (Figure 2d). Similarly, FLICA assays confirmed increased caspase-1 activation (4-fold) in Dox-treated cells (Figure 2e). Immunofluoresence staining of caspase-1 in the cardiac HL1 cell line (Figure 2f) and in primary cardiomyocytes (Figure 2g) showed caspase-1 localization to the nucleus in Dox-treated cells.

**Figure 3:**
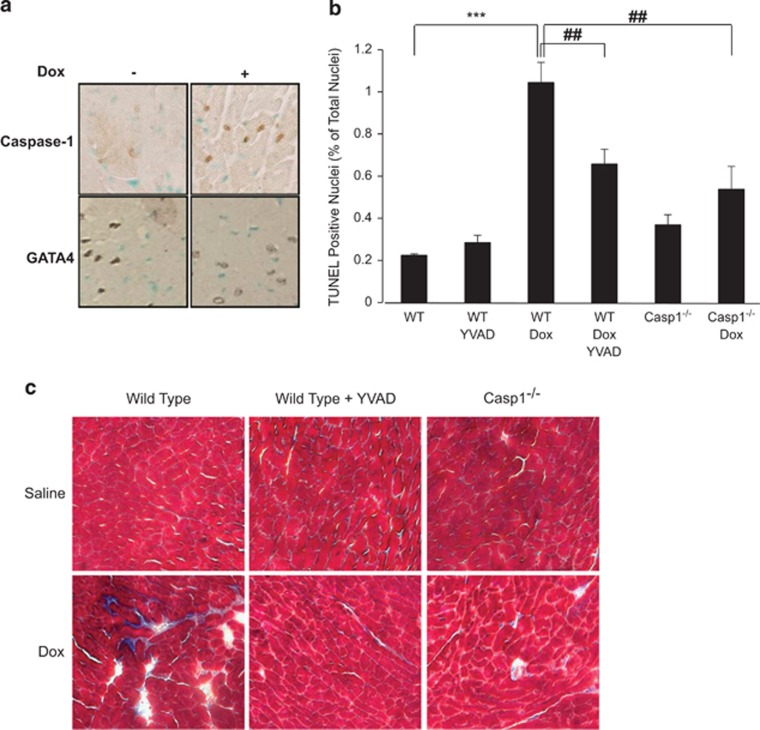
Caspase-1 inhibition is protective against Dox cardiotoxicity *in vivo*. (**a**) Dox induces an increase in caspase-1 and a decrease in GATA4 staining *in vivo*. Immunohistochemistry of ventricular tissue sections from wild-type mice treated with Dox or vehicle. Caspase-1 staining is shown in the top panels and GATA4 staining in the bottom panels. (**b**) Caspase-1 inhibition or loss attenuates cardiomyocyte cell death *in vivo*. Quantification of TUNEL assays of wild-type mice treated with Dox and YVAD-CHO as well as Casp1^−/−^ mice treated with Dox. The results are shown as the mean±S.E.M. and analyzed by Student's *T*-test of wild-type control mice (*) or of wild-type Dox-treated mice (^#^). ***P≤0.0001, ^##^*P*≤0.001. (**c**) Effect of caspase-1 inhibition or loss on Dox induced cardiac fibrotic cardiac lesions *in vivo*. Trichrome staining of transverse sections of left ventricular tissue of wild-type mice treated with Dox and YVAD-CHO or Casp1^−/−^ mice treated with Dox. Blue staining represents fibrotic lesions

Caspase-1 nuclear localization in response to Dox was also observed *in vivo*. Wild-type mice treated with Dox show stronger nuclear caspase-1 staining in comparison with control mice and a concomitant decrease in GATA4 nuclear staining (Figure 3a). To determine the effect of caspase-1 inhibition on cardiomyocyte cell death and cardiac remodeling, Terminal Deoxynucleotidyltransferase- Mediated dUTP End-Labeling (TUNEL) assays and trichrome staining were carried out on heart tissue sections of wild-type mice treated with Dox in the presence or absence of the caspase-1 inhibitor YVAD-CHO (Figures 3b and c). Treatment with YVAD-CHO significantly reduced the number of TUNEL-positive nuclei and fibrotic lesions, consistent with a role for caspase-1 in Dox-induced cardiotoxicity. Casp1^−/−^ mice treated with Dox showed an attenuated response compared with similarly treated wild-type mice as measured by cell death and the presence of fibrosis (Figures 3b and c). These results suggest that reduction of caspase-1 activity *in vivo* is protective against Dox cardiotoxicity.

**Figure 4:**
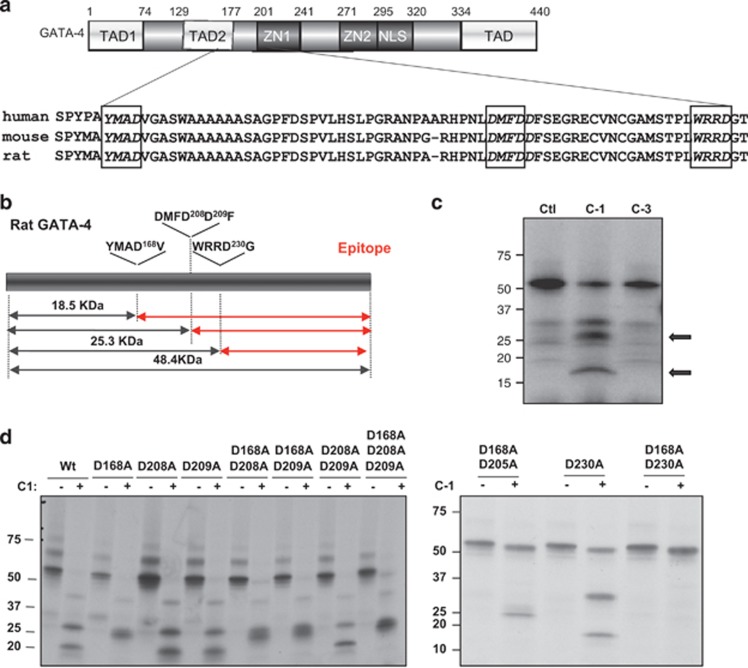
GATA4 is a direct substrate for caspase-1. (**a** and **b**) Schematic representation of rat GATA4 (accession number P46152). Alignment of GATA4 from different species shows that the putative cleavage sites YMAD168, DMFD208 and WRRD230 in boxes are conserved in human, mouse and rat. (**b**) Predicted size of GATA4 fragments cleaved by caspase-1. The depicted red fragments can be detected by the GATA4 antibody (epitope). (**c**) *In vitro* caspase cleavage assays. *In vitro* translated radiolabelled GATA4 was exposed to purified caspase-1 (C-1) and caspase-3 (C-3). Arrows indicate cleavage products by caspase-1 but not by caspase-3. (**d**) Caspase-1 cleavage of GATA4 mutants identifies D168 and D230 as cleavage sites. *In vitro* cleavage assays using purified caspase-1 and *in vitro* translated GATA4 WT and GATA4 mutants (single or double mutations as indicated in **d**). Note how double mutation of D168 and D230 prevents the cleavage by caspase-1

**Figure 5:**
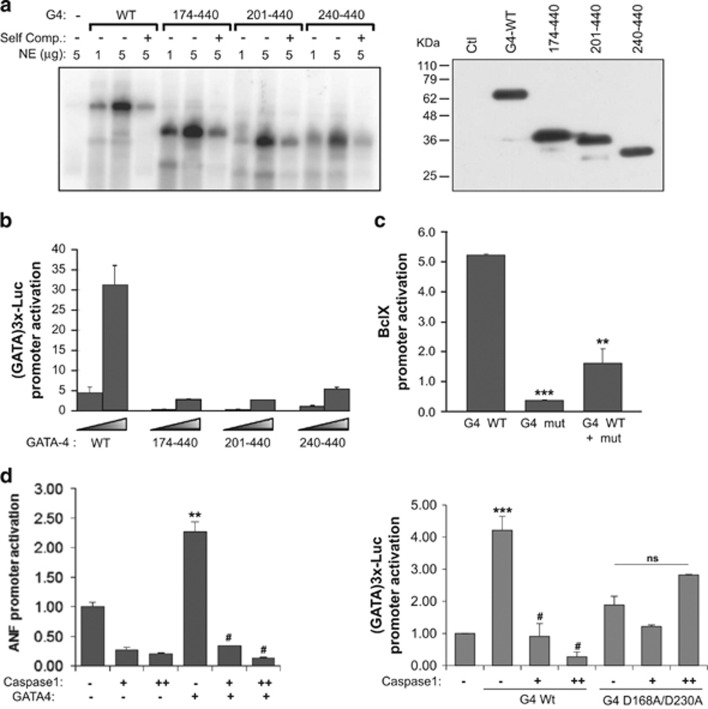
Caspase-1 is a negative regulator of GATA4 transcriptional activity. (**a**) Left panel. N-terminal truncated GATA4 proteins bind DNA. Nuclear extracts from AD293 cells transfected with the indicated GATA4 constructs were tested for their ability to bind GATA elements using EMSAs. NE: nuclear extracts, self comp: self competition with cold probe. The right panel is a western blot showing equivalent protein expression levels for all constructs. (**b**) Dose response for wild-type and truncated GATA4 protein co-transfected with the (GATA)3x-Luc promoter. (**c**) Caspase-1 cleaved GATA4 acts as dominant negative. GATA4 WT and GATA4 mut (aa 174–440) were co-transfected with the BclxL promoter. The results are shown as mean±S.E.M. and analyzed by one-way ANOVA with Bonferroni post-test relative to the GATA4 WT treatment. **P≤0.01, ***P≤0.001. (**d**) Effect of caspase-1 on GATA4-dependent transcription. Left panel: (GATA)3x–luc reporter (1 *μ*g) was co-transfected with 100 ng of native or mutant GATA4 expression vector with or without 50 and 500 ng of caspase-1 expression vector. The results are shown as mean±S.E.M. and analyzed by one-way ANOVA with Bonferroni post-test relative to the control (*) or GATA4 WT treatment (^#^). **P≤0.01, ^#^*P*≤0.001. Right panel: Effect of caspase-1 on the ANF promoter in response to GATA4. The amount of plasmid DNA used is the same as in left panel. The data are the mean±S.E.M. of two experiments carried out in duplicate. The results are shown as mean±S.E.M. and analyzed by one-way ANOVA with Bonferroni post-test relative to the control (*), GATA4 WT treatment (^#^) or mutant GATA4 treatment. ***P≤0.001, ^#^*P*≤0.001, ns=not significant. Note how caspase-1 completely abrogates GATA4 activation and how mutation of the two major caspase-1 cleavage sites renders GATA4 resistant to this effect

To determine whether GATA4 is a direct substrate of caspase-1 or -3, we searched for putative caspase recognition motifs on the GATA4 protein. Caspase-3 preferably cleaves at DEVD sequences whereas the preferred sites of caspase-1 contain a bulky and hydrophobic amino acid at the P4 position such as tryptophan and tyrosine (e.g., W/YxxD).^13, 14^ Two putative caspase-1 sites that fit these criteria are present on the GATA4 protein and are evolutionary conserved in human, mouse and rat: YMAD^168^ within the major transcription activation domain and WRRD^230^ within the first zinc finger (Figure 4a). Another conserved motif DMFD^208^ may correspond to a low affinity caspase-3 recognition site. Figure 4b depicts the possible polypeptides resulting from caspase cleavage. Incubation of *in vitro* translated GATA4 with active caspase-1 produced three fragments around 18, 26 and 32 KDa. In contrast, no caspase-3 cleavage products were detected (Figure 4c). The fragments obtained from the caspase-1 digestion are consistent with processing cleavage at D168 and D230. To confirm that these are caspase-1 cleavage sites, we prepared a series of mutant proteins in which these residues alone or in combination are converted into alanine effectively eliminating the caspase motif. As shown in Figure 4d, mutation of both D168 and D230 render GATA4 completely resistant to caspase-1 cleavage. These results confirm that GATA4 is a direct caspase-1 substrate and that caspase-1 processes GATA4 at two specific cleavage sites. Of note, cleavage at either position would result in a truncated nuclear GATA4 protein capable of binding DNA as shown in Figure 5a, but missing the N-terminal transactivation domains. As well, cleavage at D230 would lead to loss of the N-terminal zinc finger, a region critical to protein–protein interactions.^15; 16^ As expected, the deletion mutants that would result from cleavage at D168 and D230 had reduced transcriptional activation (Figure 5b) and when co-expressed with native GATA4, reduced its activity on target promoters (Figure 5c).

Next, we tested the effect of caspase-1 on GATA4 activity *ex vivo*. NIH3T3 cells were co-transfected with GATA4 and a GATA-dependent reporter in the presence or absence of caspase-1. As shown in Figure 5d, caspase-1 dose dependently inhibited GATA4 transcriptional activity of the reporter. A similar effect was also observed on the ANF promoter, a well-known GATA4 target. In contrast, the activity of a caspase-1-resistant GATA4 mutant (D168A/D230A) was not significantly affected by caspase-1. These results indicate that GATA4 is a caspase-1 substrate and that caspase-1 is a negative regulator of GATA4.

**Figure 6:**
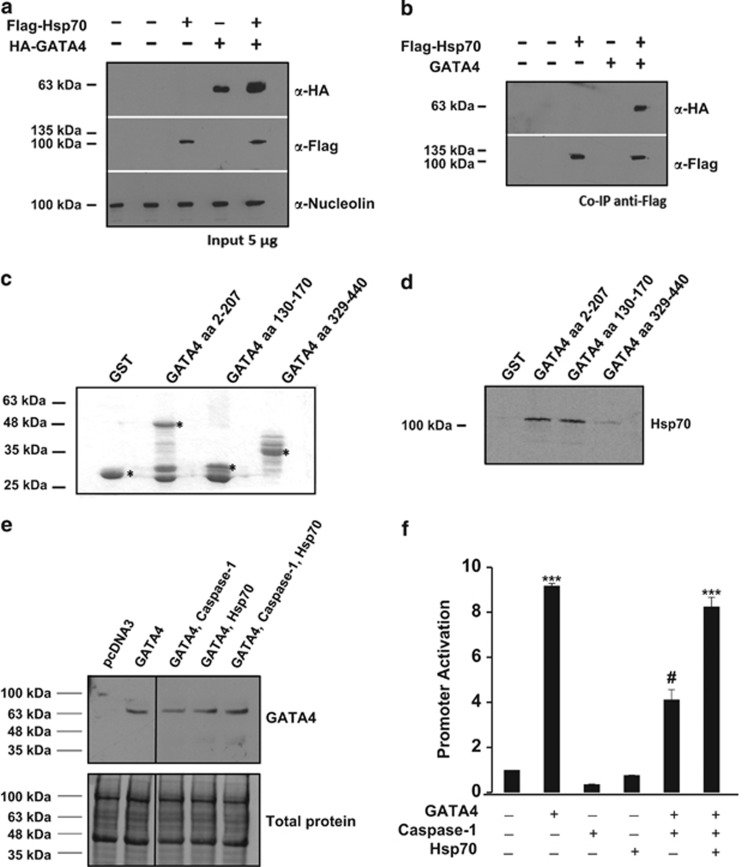
HSP70 physically interacts with GATA4 and rescues caspase-1 inhibition. (**a** and **b**) HSP70 co-immunoprecipitates with GATA4. Nuclear extracts from AD293 cells transfected with HA-GATA4 and/or Flag-HSP70-GFP were immunoprecipitated with anti-Flag antibody, separated on a 10% (vol/vol) SDS-PAGE and immunoblotted with anti-HA, anti-Flag and anti-Nucleolin antibodies. (**c** and **d**) HSP70 interacts directly with the N-terminal of GATA4. GST and GST bound GATA4 aa 2–207, aa 130–170 and aa 329–440 fusion proteins were incubated with *in vitro* translated HSP70. Bound proteins were resolved using SDS-PAGE (12% vol/vol) and revealed using autoradiography. Fusion protein inputs (**c**) were resolved using SDS-PAGE (12% vol/vol) and stained using coomassie blue. Astrices indicate fusion protein bands. (**e**) HSP70 prevents caspase-1 mediated GATA4 processing. NIH 3T3 cells were transfected with expression vectors for GATA4, caspase-1 and/or HSP70. Nuclear extracts were analyzed by western blot. (**f**) Hsp70 rescues caspase-1 mediated inhibition of GATA4 transcriptional activity. Transfection into NIH3T3 cells of the GATA-dependent Nppb promoter –Luc reporter along with the indicated expression vectors for GATA4, caspase-1 and HSP70 and activations thereof. The results are shown as mean±S.E.M. and analyzed by one-way ANOVA with Bonferroni post-test relative to the control (*) or GATA4 treatment (^#^). ***P≤0.001, ^#^*P*≤0.001 (*n*=3). Note how caspase-1 inhibits GATA4 transactivation in the absence but not in the presence of HSP70

Inhibition of GATA4 – a cardiomyocyte survival factor – by caspase-1 is consistent with the reported involvement of caspase-1 in myocyte cell death and heart failure. We asked whether interaction of GATA4 with other cofactors might serve to mask the caspase-1 recognition motifs and protect GATA4 from caspase-1 cleavage. We focused on HSP70 because it was identified by mass spectrometry as a component of nuclear GATA4 complexes in cardiogenic TC13 cells (our unpublished data) and because HSP70 is cardioprotective.^17^ Co-immunoprecipitation of transfected GATA4 and HSP70 confirmed that the two proteins interact in cell nuclei (Figures 6a and b). Pull down assays using GST-GATA4 proteins (Figure 6c) and *in vitro* translated HSP70 were carried out to identify the HSP70 interacting domain on GATA4. As shown in Figure 6d, HSP70 bound mainly the N-terminal domain of GATA4 and a 40 amino-acid fragment spanning GATA4 amino acids 130–170 was sufficient to retain HSP70. To determine the effect of HSP70 and Caspase-1 on GATA4 protein expression, nuclear extracts from NIH3T3 cells transfected with GATA4, Caspase-1 and HSP70 were analyzed by western blot (Figure 6e). Compared with transfection with GATA4 alone, co-transfection of caspase-1 and GATA4 yielded lower levels of GATA4 protein. However, GATA4 protein levels were rescued by concomitant HSP70 expression. The relevance of this interaction on GATA4 transcriptional activity was examined by luciferase assay. We co-transfected a GATA-luciferase reporter with a GATA4 expression vector in the presence or absence of caspase-1 and HSP70 (Figure 6f). HSP70 prevented the caspase-1 mediated reduction of GATA4 transcriptional activation, maintaining GATA4 activity to a similar level as observed in the absence of caspase-1. Together, the data indicate that GATA4 is a caspase-1 substrate and suggest that physical interaction with HSP70 may protect GATA4 from caspase-1 processing and inactivation.

## Discussion

Transcription factor GATA4 is a critical survival factor for cardiomyocytes and an angiogenic factor of the infarcted heart.^6, 9, 18, 19^ Decreased levels of GATA4 promote cardiomyocyte death and sensitize myocytes to drug induced cell death. The data presented here reveal that GATA4 is inactivated by caspase-1 cleavage, which leads to transcriptional downregulation of cell survival pathways (e.g., Bcl-xL) and irreversible cardiac damage. This negative feedback loop would amplify the deleterious effects of cardiotoxic insults and is consistent with the degenerative nature of some cardiac disease such as heart failure. Conversely, the finding that HSP70 interacts with GATA4 to prevent caspase-1-dependent inhibitory effects might explain – at least in part – the cardioprotective effects of HSP70. For example, erythropoietin has been shown to have cardioprotective effects against ischemic or non-ischemic heart disease including Dox-induced cardiotoxicity.^20^ Erythropoietin prevents Dox-mediated GATA4 depletion and also increases HSP70 expression, which may serve as the first control against GATA4 depletion.^21^ The mechanisms by which erythropoietin may exert its cardioprotective role via induction of HSP70 and stabilization of GATA4 would be reminiscent of its mechanism of action in erythropoiesis where it induces HSP70 to protect against caspase-3 cleavage of GATA1.^22^ Other cardioprotective inducers such as exercise, CaMKII and preconditioning also increase HSP70.^17, 23, 24, 25^ It is therefore tempting to speculate that HSP70 cardioprotection in these instances also involves preventing caspase-1-mediated GATA4 degradation.

Caspase-1 is best known for its role in the NLRP3 inflammasome where it cleaves and processes IL-1*β* and IL-18.^1^ The involvement of the NLRP3 inflammasome has been documented in several cardiac contexts including acute myocardial infarction, heart failure and myocardial contractile dysfunction due to sepsis.^26, 27, 28^ Furthermore, Dox has also been shown to induce the NLRP3/caspase-1/IL-1*β* pathway in the context of macrophages and dendritic cells.^29^ However, our findings demonstrate that in cardiac tissue, Dox-induced caspase-1 activation is involved with other non-canonical pathways as well. This is particularly interesting given that transgenic mice overexpressing caspase-1 show an increase in cardiomyocyte cell death without a concomitant increase in IL-1*β* and IL-18 secretion.^4^ This suggests the involvement of a non-inflammatory mechanism such as cleavage of GATA4 and subsequent dysregulation of cardiomyocyte survival pathways.

Few validated caspase-1 substrates are known besides IL-1*β* and IL-18. Using a proteomic approach, 41 proteins were identified that can be cleaved by caspase-1; they include translation machinery, chaperones and cytoskeletal proteins as well as several enzymes of the glycolysis pathway.^30^ No nuclear targets for caspase-1 have yet been identified despite the fact that caspase-1 expression is observed in the nucleus.^27^ This is in contrast to caspase-3 that has been reported to cleave several transcription factors including GATA1 in hematopoietic cells and MEF2 in neuronal cells.^31, 32^ The identification of GATA4 as a nuclear substrate for caspase-1 suggests a direct role for this caspase in transcriptional regulation. Interestingly, sequence analysis reveals that the D230 recognition site is conserved in all six members of the GATA family which, in addition to the heart, have a critical role in immune cells, neurons and the gut. This is noteworthy given the role of caspase-1 in inflammation, neuronal survival and, more recently, in triglyceride metabolism.^33, 34^ Whether caspase-1 targets additional GATA proteins or other transcription factors in cardiac and extra cardiac tissues will be worth investigating.

## Materials and Methods

### Cell culture and transfections

Cardiomyocytes from 4-day-old Sprague-Dawley rats (Charles River, Wilmington, MA, USA) were harvested, cultured and manipulated as previously described.^6^ Myocytes were treated with Doxorubicin (Sigma, St. Louis, MO, USA) at 300 nM for the indicated time in the presence or absence of a protease inhibitor (MG-132, 10 *μ*M in DMSO, CalBioChem, Billerica, MA, USA, 474790) or caspase inhibitors: caspase-1 inhibitor (YVAD-CHO, 10 *μ*M in DMSO, CalBioChem 400011) or pan-caspase inhibitor (zVAD-FMK, 10 *μ*M in DMSO, CalBioChem 219007). Inhibitors were added to cardiomyocytes 30 min before the addition of Dox. NIH3T3, HL1, TC13 and AD293 cells were maintained and manipulated as previously reported.^35, 36^ Luciferase assays were carried out as described previously.^37^

### Western blot

Western blots of nuclear extracts from cardiac myocytes or other cell lines overexpressing various GATA4 proteins were performed as previously described.^8^ Western blots of nuclear extracts from cardiac myocytes or other cell lines overexpressing various GATA4 proteins were performed as previously described.^8^ Anti-HA (Santa Cruz, Santa Cruz, CA, USA, sc-805) anti-Flag (Sigma, F1804), anti-p300 (Santa Cruz, SC-585X) and anti-nucleolin (Santa Cruz, sc-55486) were all used at a dilution of 1/500. Anti-caspase-1 (Cell Signaling, Danvers, MA, USA, 2225), antiBclxL (Cell Signaling, 2762), anti-GAPDH (Abcam, Cambridge, UK, ab8245) and anti-GATA4 (Santa Cruz, sc-25310) were used at 1/1000 dilution. Homemade rabbit GATA4 and GATA6 antibodies were used at a dilution of 1/2000 and 1/500, respectively.^38^

### Electrophoretic mobility shift assays

DNA binding of GATA4 mutants was assessed using nuclear extracts from AD293 cells and the proximal GATA site from the rat Nppa promoter as described previously.^8^

### Coimmunoprecipitation

AD293 cells were transfected with pCGN-HA-GATA4 and/or Flag- pcDNA3.1-F-HSP70-GFP using Effectene transfection reagent (Qiagen, Hilden, Germany, 301425) according to the manufacturer's guidelines. Nuclear extracts were incubated with anti-Flag M2 coupled magnetic beads (Sigma) overnight as described by Morin *et al.*^37^ Bound proteins were revealed with anti-HA or anti-Flag antibodies by western blot.

### TUNEL assay for apoptosis

Apoptosis was detected by the TUNEL technique as recommended in the Apoptag kit (Millipore, Billerica, MA, USA, S7100). An average of 10 random fields with 100 nuclei per field was analyzed.

### Immunofluorescence

Immunofluorescence experiments were carried out as described previously.^8^ Anti-Caspase-1 (Abcam, ab-1872) was used at a dilution of 1/200 and Alexa Fluor 546 Goat Anti-Rabbit IgG (Life Technologies, Carlsbad, CA, USA, A-11035) was used at a dilution of 1/500. Hoechst (Life Technologies, H1398) was used at a dilution of 1/5000. Images acquisition was completed using the Zeiss AxioObserver D1 microscope (Oberkochen, Germany).

### Immunohistochemistry

Immunohistochemistry was completed as previously described.^6^ Rabbit anti-caspase-1 antibody (Abcam, ab-1872) was used at a dilution of 1/200. A homemade rabbit anti-GATA4 antibody was used at a dilution of 1/500.

### *In vitro* translation and pull down assays

*In vitro* translation and pull down assays were carried out as described previously.^8^ ^35^S-labelled *in vitro* translated proteins were produced using the T7 Quick-Coupled Transcription/Translation System (Promega, Madison, WI, USA) according to the procedures provided by the manufacturer. Pull down assays were carried out as described previously.^8^ Briefly, recombinant GST-fused proteins were produced in BL-21 *E. coli* and purified on sepharose beads. *In vitro* translated proteins were incubated with GST fusion proteins overnight at 4 ^o^C. Bound proteins were detected by autoradiography.

### Caspase cleavage assays

Cleavage of the *in vitro* transcribed and translated ^35^S-labeled substrates was performed in a 20-*μ*l reaction containing 2 *μ*l of *in vitro* transcribed and translated ^35^S-labeled substrates by incubation at 37 °C for 4 h in the presence or absence of purified human recombinant caspase-1 or caspase-3 (170 ng) in CHEG buffer (with 10 mM dithiothreitol freshly added). The cleavage reaction was terminated by the addition of Laemmli SDS loading buffer and resolved by SDS-PAGE. The gel was fixed in 10% acetic acid and 40% ethanol for 0.5 h; the signal was then amplified by incubating the gel with NAMP 100 V amplifying solution (Amersham Biosciences, Little Chalfont, Buckinghamshire, UK) for 30 min The gel was placed on a Whatman paper, dried at 70 °C for 1 h, and exposed at −80 °C, and the signal was viewed by autoradiography.^30^

### Real-time PCR

RNA was extracted using Trizol and then reverse transcribed with the Omniscript reverse transcriptase (Qiagen). QPCR analyses were used to measure change in GATA4 and ribosomal protein S16 mRNA levels using the Quantitech SYBR green (Qiagen).

### Mice

C57/B6 mice were treated with Dox as previously described.^6^ Casp1^−/−^ mice have been previously described.^39^ For Dox and YVAD-CHO experiments, animals were injected i.p. with 5 mg/kg YVAD-CHO and 20 mg/kg Dox. Injections were separated by 1 h. After 1 week, animals were killed by cervical dislocation and the heart was cryopreserved. All experiments were approved by the University of Ottawa and McGill University animal care committees and were carried out as per institutional guidelines for animal care. Mason trichrome staining was completed as previously described.^40^

### FAM-FLICA assay

The FAM-FLICA assay is specific to active caspase-1 and measures binding of caspase-1 to cognate sites. The assays were done as per the manufacturer's instructions (ImmunoChemistry Technologies, catalog number 97, Bloomington, MN, USA). Briefly, cardiomyocytes plated on glass coverslips were incubated with FAM-FLICA reagent diluted in serum-free media for 1 h at 37 ^o^C. Cells were then washed three times for 5 min in media and fixed with 4% PFA. Cells were then washed three times in PBS and mounted with Prolong Gold (Life Technologies, P36930). Fluorescence image acquisition was completed using the Zeiss AxioObserver D1 microscope.

### Plasmids

GATA4 and all luciferase reporters used were previously described.^6, 41^ GATA4 point mutations were subcloned into the pGEX vector and the N-terminal flag-tagged Caspase-1 constructs were produced by PCR from rat cDNA and was subcloned into the pcDNA3 vector. The pcDNA3.1-F-HSP70-GFP construct was a kind gift from Dr Stephen Lee (University of Ottawa).^42^ All constructs were verified by sequencing.

## Abbreviations

Casp1^−/−^: caspase-1 knockout mice
Dox: doxorubicin
FLICA: fluorescent labeled inhibitor of caspases
HSP70: heat-shock protein 70
IL-1*β*: interleukin- 1*β*
IL-18: interleukin-18
YVAD-CHO: a cell permeable caspase-1 inhibitor
zVAD-CHO: a cell permeable pan-caspase inhibitor
